# The association between cognitive impairment/dementia and albuminuria: a systematic review and meta-analysis

**DOI:** 10.1007/s10157-021-02127-3

**Published:** 2021-08-28

**Authors:** Hongqin Li, Shuailin Zhao, Ruiyu Wang, Baoshan Gao

**Affiliations:** 1 Department II of Urology, First Hospital of Jilin University, No. 71 Xinmin Street, Changchun, 130021 Jilin China; 2 School of Pharmaceutical Science, Jilin University, Changchun, Jilin China

**Keywords:** Albuminuria, Cognitive impairment, Dementia, Meta-analysis

## Abstract

**Background:**

To identify the association between albuminuria and dementia or cognitive impairment.

**Methods:**

The literature search was performed to identify relevant scientific studies through August 2019, including PubMed/Medline and EMBASE. For inclusion, the studies had to fulfil the following criteria: population-based cohort, case–control or cross-sectional studies; quantifying an association of albuminuria with cognitive impairment or dementia; and reported odds ratio (OR), and the corresponding 95% confidential interval (95% CI). Random effects model was used to yield pooled estimates.

**Results:**

A total of 16 studies (11 cohort studies and five cross-sectional studies) were included in the meta-analyses. Based on the fully adjusted estimates, albuminuria was associated with a significant higher risk of cognitive impairment or dementia. Furthermore, the same trend existed for cognitive impairment and dementia, respectively. In addition, both of Alzheimer’s diseases (AD) and vascular dementia (VaD) were significantly associated with albuminuria.

**Conclusion:**

Albuminuria was significantly associated with cognitive impairment and dementia. Corresponding to an earlier subclinical time-point in kidney disease progress, albuminuria may be a potential factor predicting the future occurrence of dementia.

**Supplementary Information:**

The online version contains supplementary material available at 10.1007/s10157-021-02127-3.

## Introduction

There is an increasing number of people diagnosed with dementia worldwide, comprising major public health concerns [1]. Among individuals aged more than 60 years old, 5–7% of them are estimated to suffer from dementia, whereas the prevalence of mild cognitive impairment (MCI) is approximated to 20–30% [2, 3]. The identification of modifiable risk factors of cognitive decline or dementia is of vital importance to develop preventive strategies [4, 5].

As a marker of renal microvascular disease, albuminuria often occurs in the presence of hypertension and diabetes mellitus (DM), and shares common risk factors with dementia, including increasing age, elevated systolic blood pressure (SBP), and increased levels of inflammation factors [6, 7]. Previous studies has identified albuminuria as a potential risk factor for dementia and cognitive impairment [8–10]. Furthermore, Georgakis et al. had conducted a meta-analysis and identified that albuminuria was independently associated with cognitive impairment, dementia and cognitive decline in 2017 [11]. Adding newly reported data, this meta-analysis aimed to further clarify the association between albuminuria and dementia or cognitive impairment.

## Materials and methods

### Literature search

The literature search of computerized databases was performed to identify relevant scientific studies through August 2019, including PubMed/Medline and EMBASE. Two search themes were combined with the Boolean Operator “And”. The first Boolean search used the terms of “albuminuria”, “proteinuria” or "kidney disease", and the second theme was cognitive impairment using subject headings, including “dementia”, “cognition”, “cognitive impairment”, or “Alzheimer’s disease”. In addition, the reference lists of identified studies were scanned to enhance the searches. This meta-analysis was designed, conducted and reported following the pre-determined protocol in accordance with the Meta-analysis of Observational Studies in Epidemiology (MOOSE) reporting guidelines [12].

### Study selection

Hongqin Li and Baoshan Gao independently assessed the eligibility of each searching result. Full texts of selected articles were reviewed to further identify eligible studies included in the study, after screening all the potential articles with reading titles and abstracts. The disagreement was resolved by discussion. For inclusion, the study had to meet the following criteria: adult participants with aged ≥ 18; longitudinal studies (cohort or case–control) or cross-sectional studies; quantifying an association of albuminuria with cognitive impairment or dementia; and reported odds ratio (OR), and the corresponding 95% confidential interval (95% CI). Studies with (1) involvement of solely children/adolescents and referring exclusively to hemodialysis patients, or patients with chronic autoimmune disorders or HIV-infected, (2) in vitro and animal studies, (3) reviews, case reports, abstracts, editorials, and comments would be excluded.

### Data extraction and quality assessment

Shuailin Zhao and Ruiyu Wang extracted data from all eligible studies respectively, with any disagreement resolved by consensus. For each relevant study, the following data were extracted: the first author’s last name, year of publication, study design, geographic location, sample size, median age, gender composition, clinical characteristics (body mass index [BMI], diabetes mellitus [DM], hypertension, cardiovascular disease [CVD], blood pressure [BP], albuminuria, estimated glomerular filtration rate [eGFR], cholesterol levels), exposures, outcome assessment, and analysis results. We adopted an evaluation system based on Newcastle–Ottawa scale (NOS) to assess the quality for both longitudinal and cross-sectional studies [13].

### Statistical analysis

Albuminuria was defined by albumin-to-creatinine ratio (ACR) or 24-h urinary albumin excretion; cut-off points of either (≥ 30 mg/gCr or mg/24-h, respectively), or (gender-specific ≥ 17 in men and ≥ 25 mg/gCr or mg/24-h in women, respectively) were used [14]. Microalbuminuria was defined as excretion of 30–300 mg/24 h of albumin, whereas macroalbuminuria was defined as excretion of more than 300 mg/24 h of albumin.

Two major outcomes were examined, including dichotomized cognitive impairment/ dementia outcomes and continuous cognitive function measures. The first included cognitive impairment, defined by validated instruments, and dementia based on clinical diagnostic criteria. Various validated neuropsychological tests were used to evaluate continuous cognitive function. The diagnosis of dementia, and classification of Alzheimer’s diseases (AD) and vascular dementia (VaD) were based on clinical diagnostic criteria.

The ORs were used as the common measure of association, and both relative risks (RRs) and hazard ratios (HRs) were considered equivalent to ORs [15]. ORs and 95% CIs for the effect of albuminuria on the risk of cognitive impairment and dementia were pooled, implemented with random-effects models in meta-analyses. The heterogeneity among the studies was estimated with the *Q* test and *I*^2^ statistics [16]. Heterogeneity exists if *P* value of *Q* test < 0.05. We had stratified the AD and VaD for further analysis. Subgroup analyses by study design were carried out, to assess the potential influence on the association. Publication bias was assessed through the Egger’s test and funnel plots [17, 18]. All analyses were done with Stata software version 12 (StataCorp LP, College Station, TX, USA).

## Results

### Literature search and study characteristics

**Fig. 1:**
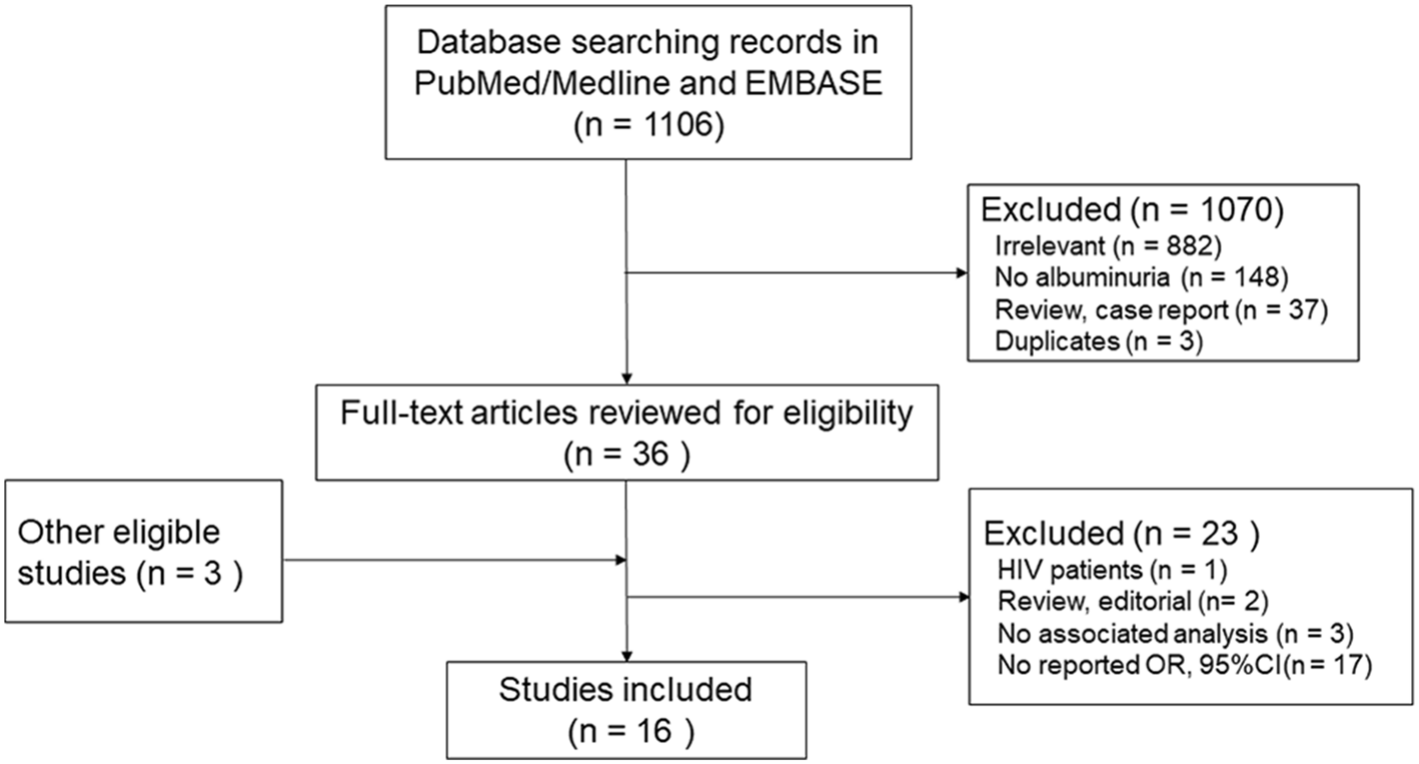
Flow diagram

Our initial search yielded a total of 1106 unique citations, of which 36 articles were considered to be potentially relevant and identified to retrieval with full-text review (Fig. 1). Another 23 articles were further excluded for the following reasons: association not evaluated (*n* = 3); no ORs/HRs or the corresponding 95% CI (*n* = 17); review or editorial (*n* = 2) and HIV patients (*n* = 1). Another three eligible studies were identified through references of relevant publications.

Finally, 16 studies (11 longitudinal and five cross-sectional studies), with a total of 127,296 participants (5488 cases of cognitive impairment, and 1266 cases of dementia) were included in the meta-analysis [19–34]. Among all the 16 studies, six studies explored the association with dementia and five of them made a stratification analysis on AD and VaD [21, 22, 26, 30–32]. One study only contained the analysis with a continuous measure of cognitive impairment. The majority of studies provided results of multivariable analyses, adjusting major confounders, including age, sex, estimated glomerular filtration rate (eGFR), cardiovascular disease, diabetes, and hypertension. The average age for the participants ranged from 50 to 78 across all studies. Table 1 provides the detailed characteristics of all the included population-based studies, including the NOS scale.

**Table 1:** Characteristics of population-based studies assessing the relation between albuminuria and dementia or cognitive impairment

Authors	Year	Country	Design	Population	Mean age	Female (%)	Cognitive test/Diagnosis	Albuminuria assessment	Adjustment for confounders	Follow-up (years)	NOS
Barzilay	2008	US	Longitudinal	2316	/	59	Dementia	ACR ≥ 30	Age, sex, race, education, history of CHD, stroke, hypertension, diabetes, smoking, serum cholesterol, LDL, CRP, eGFR, APOEε4	> 5	8/9
Abbatecola	2008	Italy	Cross-sectional	140	78	/	MMSE	Continuous log ACR	Baseline MMSE, age, education, BMI, smoking status, depression, drug intake, CV-PPG, SBP, IMT	/	8/10
Vupputuri	2008	US	Cross-sectional	2386	71	66	DSS score 0–32	ACR ≥ 30	Age, ethnicity, gender, education, smoking, diabetes, hypertension, total cholesterol, HDL, CHD, CHF, MI, stroke, anemia, CRP	/	9/10
Chen	2008	China	Cross-sectional	175	61	51	MMSE	ACR	Age, education, BG, cholesterol, history of MI, stroke, smoking, BP		7/10
Weiner	2009	US	Cross-sectional	335	73	73	Executive functioning*	Macroalbuminuria ACR > 250 (M) or 350 (F)	Sex, race, diabetes, cardiovascular disease, hypertension, current use of ACEI or ARB, and eGFR	/	7/10
Wang	2010	China	Longitudinal	1351	59	48	MMSE	ACR > 25 (F) or 17 (M)	Age, gender and education	4	7/9
Barzilay	2011	International	Longitudinal	28,384	67	29	MMSE 3-Point or Greater Decrease	Microalbuminuria: ACR: 30–299	Age, sex, ethnicity, education, history of CVD, DM, hypertension, baseline SBP, smoking, BMI, and eGFR, alcohol use, exercise, depression, and medication use	5	8/9
Tamura (a)	2011	US	Longitudinal	19,339	64	60	Item S1	Microalbuminuria: ACR: 30–299	Age, race, sex, education, region, diabetes, hypertension, CVD, stroke, smoking alcohol use, eGFR	4	8/9
Tamura (b)	2011	US	Longitudinal	3591	58	47	3MS	ACR four quartiles	demographics and Vascular Risk Factors	NA	6/9
Helmer	2011	France	Longitudinal	1003	74	61	MMSE	ACR > 30	age, sex, educational, APOE4 genotype, hypertension, CVD, hypercholesterolemia, hypertriglyceridemia, stroke, diabetes, smoking, BMI, and baseline eGFR	7	7/9
O’Hare	2012	US	Longitudinal	2968	74	60	DSM-IV criteria	Proteinuria (positive, trace, no)	Time-varying indicator variables	6	7/9
Barzilay	2013	US	Longitudinal	2957	63	47	≥ 5% decline in DSST	ACR ≥ 30	Age, sex, race, education, alcohol consumption, BMI, SBP, secondary CVD prevention, LDL, baseline eGFR	4–6	7/9
Higuchi	2015	US	Longitudinal	3583	78	/	DSS score 0–33	Proteinuria (positive, trace, no)	Age, education, APOEε4, stroke, hypertension, SBP, DM, fasting BG, physical activity index, baseline cognitive abilities screening instrument score and time of follow-up	8	7/9
Wei	2016	US	Cross-sectional	1982	70	54	DSST	Urinary albumin	Age, sex, race/ethnicity, poverty status, education, physical activity, BMI, cigarette smoking, and alcohol consumption	/	9/10
Takae	2018	Japan	Longitudinal	1562	71	48	Dementia	ACR ≥ 30	Age and sex, educational, history of stroke, SBP, antihypertensive agents, DM, total cholesterol, BMI, smoking, alcohol, and exercise, log eGFR	10	7/9
Gabin	2019	Norway	Longitudinal	48,508	50	54	Dementia	ACR four quartiles	ACR, age, sex, education, GFR, cholesterol, non-fasting BG, serum iron, BMI, pulse, history of MI, DM, angina, stroke, smoking, subjective health status	> 10	8/9

*ACR* albumin/creatinine (mg/g); *CVD* cardiovascular disease; *SBP* systolic blood pressure; *BMI* body mass index; *LDL* low-density lipoproteins; *eGFR* estimated glomerular filtration rate; *BG* blood glucose; *DM* diabetes mellitus; *CHD* coronary heart disease; *CHF* congestive heart failure; *MI* myocardial infarction; *CRP* C-reactive protein; *CV-PPG* coefficient of variation-postprandial glucose;* IMT* intimal media thickness; *ACEI* ACE inhibitors; *ARB* angiotensin receptor blockers; *NOS* Newcastle–Ottawa scale

*MMSE* Mini-Mental State Examination; *DSS score* the digit symbol substitution score; *Item S1* the Six-item Screener; *3MS* the Modified Mini-Mental State Exam; *DSM-IV* Diagnostic and Statistical Manual of Mental Disorders, Fourth Edition; *DSST* the Digit Symbol Substitution Test

*The Mini-Mental State Examination (MMSE) and the North American Adult Reading Test (NAART) were administered to determine study eligibility. The neuropsychological test battery included multiple tests to assess a broad range of cognitive functioning is available at www.ajkd.org

### Meta-analysis

**Fig. 2:**
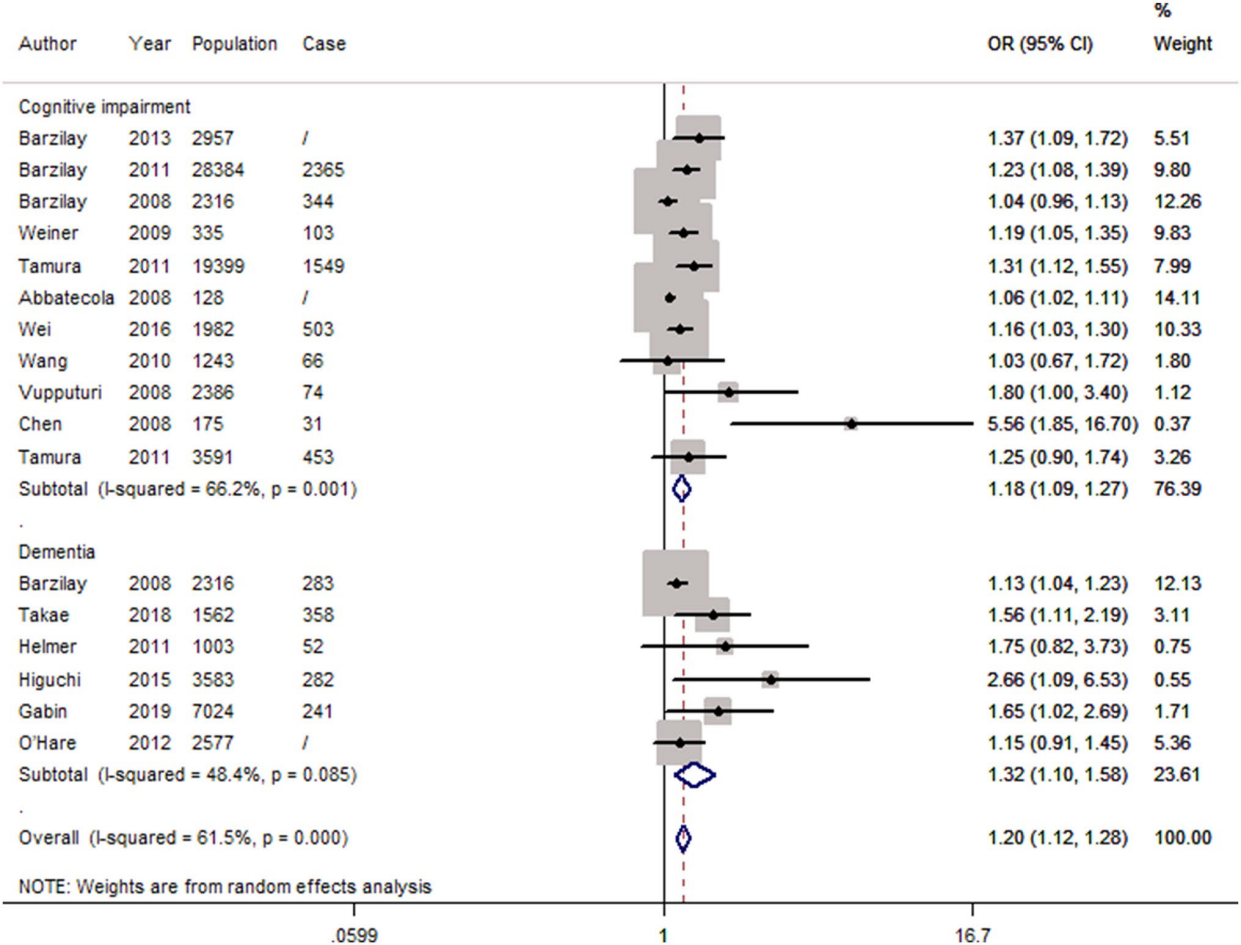
Forest plot of population-based studies assessing the relation between albuminuria and dementia or cognitive impairment (fully adjusted estimates)

**Fig. 3:**
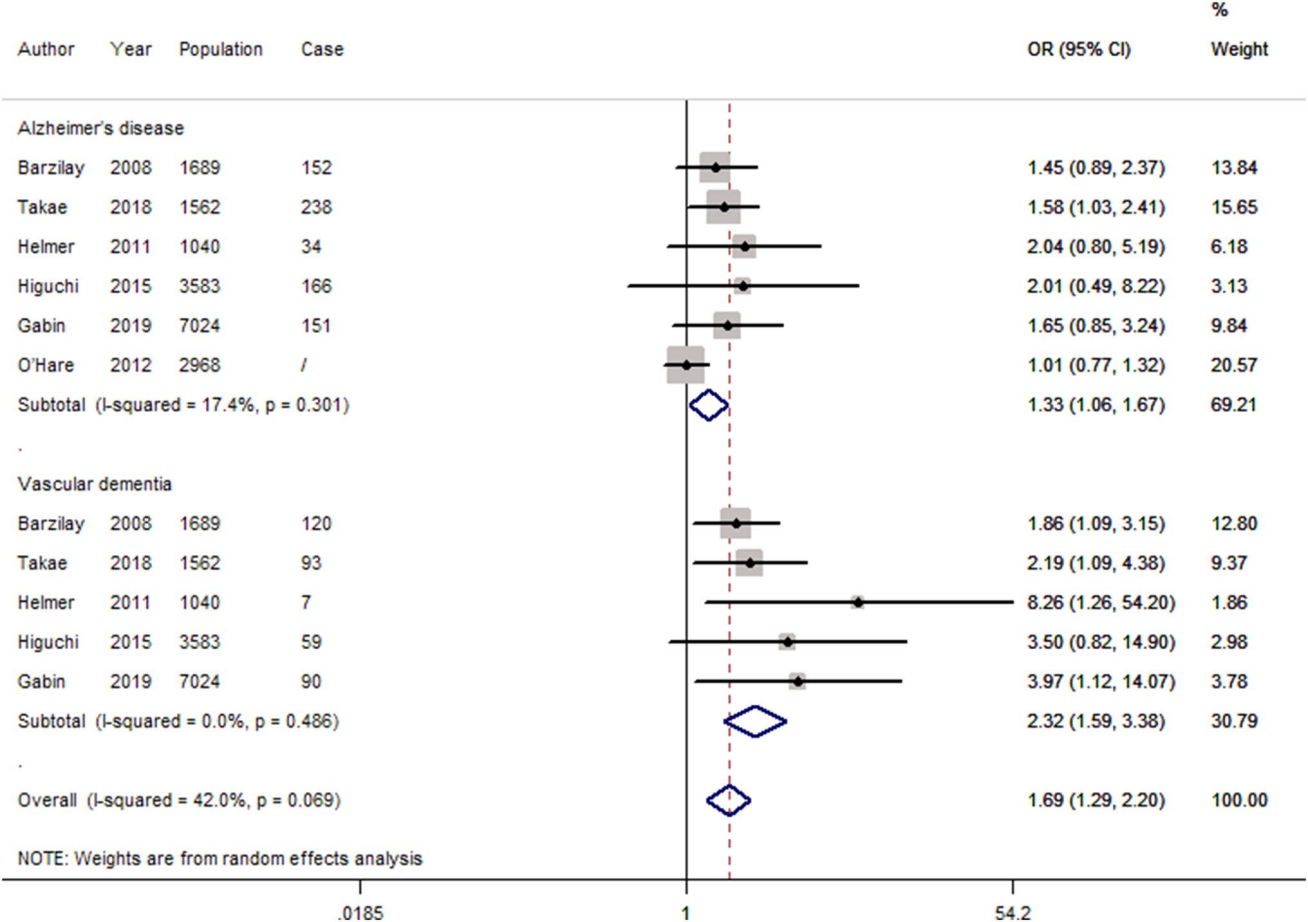
Forest plot of studies assessing the relation between albuminuria and dementia, stratified by AD and PD (fully adjusted estimates)

Based on the fully adjusted estimates, albuminuria was associated with a significant higher risk of cognitive impairment or dementia (OR 1.20, 95 CI 1.12–1.28; *P* < 0.05) as shown in Fig. 2. Furthermore, the same trend existed for cognitive impairment (OR 1.18, 95 CI 1.09–1.27; *P* < 0.05) and dementia (OR 1.32, 95 CI 1.10–1.58; *P* < 0.05), respectively. In addition, both of AD and VaD were significantly associated with albuminuria (Fig. 3, AD: OR 1.33, 95 CI 1.06–1.67; *P* < 0.05, and VaD: OR 2.32, 95 CI 1.59–3.38; *P* < 0.05). After excluding 5 cross-sectional studies, the pooled estimates for the remaining cohort studies revealed a significant association between albuminuria and cognitive impairment or dementia (Supplementary Fig. 1).

### Heterogeneity

There was significant heterogeneity for all studies included in this meta-analysis (*I*^2^ = 61.5%, *P* < 0.001). In addition, significant heterogeneity was identified for studies on cognitive impairment (*I*^2^ = 66.2%, *P* = 0.001), whereas there was mild-to-moderate heterogeneity for studies with outcomes of dementia (*I*^2^ = 48.4%, *P* = 0.085).

### Publications bias

**Fig. 4:**
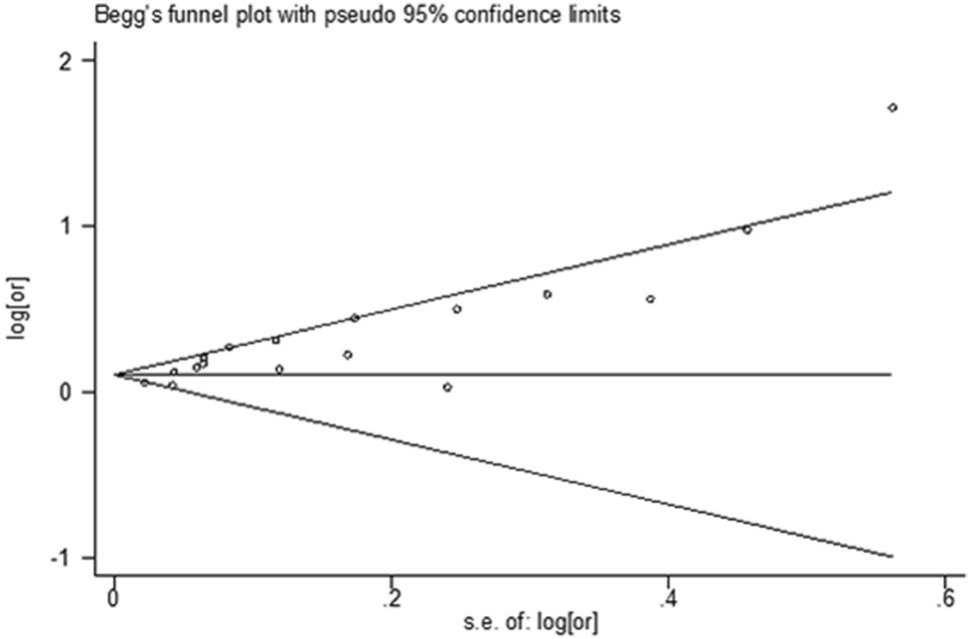
Funnel plot

The funnel plot showed an obvious asymmetry (Fig. 4). And the Egger’s test also showed evidence of publication bias (*P* < 0.01).

## Discussion

This systematic review and meta-analysis suggested that individuals with albuminuria had significantly a higher risk of cognitive impairment or dementia. Stratifying by study design, the pooled results for all cohort studies revealed the same trend between albuminuria and risk of cognitive impairment or dementia.

Our findings were consistent with the previous meta-analysis that albuminuria was independently associated with cognitive impairment and dementia [11, 35]. As an increasing prevalence of dementia worldwide, the identification of modifiable risk factors of cognitive decline or dementia is of vital importance. However, previous evidence had a high heterogeneity due to various measurements, different study design and statistical analysis. In this meta-analysis, we had a more restricted selection criteria, that only studies quantified an association between albuminuria and cognitive impairment or dementia and reported OR and 95% CI were included to better evaluate the association. In addition, two studies based on large prospective registry, with a least of 10 years follow-up in Japanese and Norway population and stratified analysis of dementia subtypes, were added to update the evidence on albuminuria and cognitive impairment/dementia.

Although the mechanism underlying the relationship between albuminuria and dementia remained undermined, several plausible mechanisms may account for the association. First, proteinuria may act as a surrogate marker of oxidative stress, which plays an important role in the pathogenesis of dementia [36]. Paragh et al. found that defect in HDL-associated antioxidant capacity played a role in the pathogenesis of AD and VaD [37]. In addition, the process of atherosclerosis, often developing for several decades due to endothelial damage, may contribute to neurodegeneration related to AD [38]. Besides, atherosclerosis associated diseases, including hypertension and DM were proven to be associated with poor performance on cognitive function test, and an increased risk of dementia [39, 40]. Furthermore, microvascular pathology, correlated with increased risks of various vascular diseases in kidney, heart and brain, may participate in the pathogenesis of VaD, and possibly AD. Autopsy data showed that microvascular pathology was associated with not only VaD, but also AD and all-cause dementia [41].

The association of albuminuria and dementia probably indicates concurrent pathology in kidneys and brain. Although the mechanism is not fully understood, evidence has proven the association of chronic kidney disease and cognitive dysfunction [42, 43]. However, those studies mainly focused on advanced kidney damage stages [44, 45]. Microalbuminuria, corresponding to an earlier subclinical time-point in kidney disease progress, could work as a more sensitive marker.

As an important method to reveal risk trends, meta-analysis provides a more precise risk estimates. In addition, most of the studies included in our meta-analysis adopted a cohort design, and the estimates with cohort studies separately were calculated, which might reduce the possibility of recall and selection bias. Moreover, the included studies contained various ethnicity, which might increase the generalizability of the findings. However, several limitations of this meta-analysis should be acknowledged. First, since only observational studies were obtained, there was a possibility that other factors might account for the association. In addition, compared with studies on dementia, the heterogeneity for studies on cognitive impairment was significant. Several methodologic issues might contribute to the substantial heterogeneity observed in the meta-analyses for cognitive impairment populations, including: (1) different criteria to measure albuminuria; (2) variation in study population (e.g. ethnicity, age range, concurrent medication, and comorbidity); (3) adjusting different confounders; and (4) different assessment tools of cognitive functioning, and one study only having continuous measures of cognition. Last, the results of the meta-analysis should be take cautiously since significant publication bias was revealed and the direct exchange of HR/RR to OR. Further evidence are warranted to verify our findings.

In summary, albuminuria was significantly associated with cognitive impairment and dementia. Corresponding to an earlier subclinical time-point in kidney disease progress, albuminuria may be a potential factor predicting the future occurrence of dementia. Targeting the pathogenesis of albuminuria or treating modifiable risk factors may have clinical implications to prevent dementia, or delay disease progression.

## Supplementary Information

Below is the link to the electronic supplementary material.

Supplementary file1 Supplementary Fig. 1. Forest plot of cohort studies assessing the relation between albuminuria and dementia or cognitive impairment (fully adjusted estimates) (TIF 61 KB)

## Data Availability

The datasets used and/or analyzed during the current study are available from the corresponding author on reasonable request.
