# Supplementary figures and images for: The association between cognitive impairment/dementia and albuminuria: a systematic review and meta-analysis
Source: Clin Exp Nephrol. 2021 Aug 28;26(1):45–53. doi: 10.1007/s10157-021-02127-3 (PMC8738457; doi:10.1007/s10157-021-02127-3)

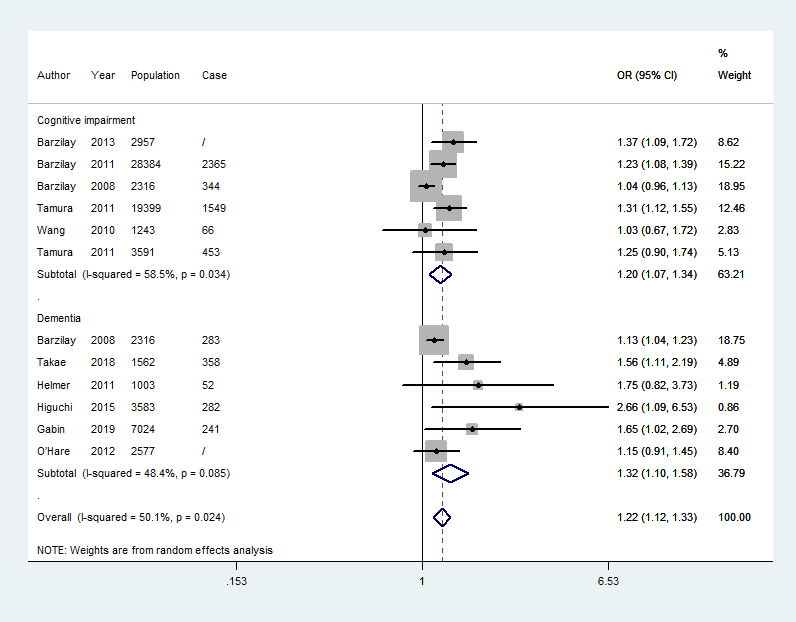

Supplement: Supplementary file 1 — Supplementary file1 Supplementary Fig. 1. Forest plot of cohort studies assessing the relation between albuminuria and dementia or cognitive impairment (fully adjusted estimates) (TIF 61 KB) [file 10157_2021_2127_MOESM1_ESM.tif]
